# Prevention of complications in neck dissection

**DOI:** 10.1186/1758-3284-1-35

**Published:** 2009-10-12

**Authors:** Cyrus J Kerawala, Manolis Heliotos

**Affiliations:** 1 Head and Neck Unit, The Royal Marsden NHS Foundation Trust, Fulham Road, London SW3 6JJ, UK; 2 North West London Regional Maxillofacial Unit, Northwick Park Hospital, Watford Road, Harrow, Middlesex HA1 3UJ, UK

## Abstract

**Background:**

The neck dissection has remained a pivotal aspect of head and neck cancer management for over a century. During this time its role has expanded from a purely therapeutic option into an elective setting, in part promoted by efforts to reduce its morbidity.

**Objectives:**

This review will consider the potential complications of neck dissection and on the basis of the available evidence describe both their management and prevention.

**Conclusion:**

Although the neck dissection continues to provide clinicians with a method of addressing cervical disease, its reliability and safety can only be assured if surgeons remain cognisant of the potential complications and aim to minimise such morbidity by appropriate management in the peri-operative period.

## Introduction

Since its original description by Crile in 1906 and subsequent popularisation by Hays Martin in 1951 the radical neck dissection (RND) remained the standard treatment for palpable or potential cervical metastasis from head and neck cancer for many decades [1,2]. This operation provides a safe and reproducible method of comprehensively addressing cervical lymph nodes. Recurrence rates after RND vary according to the bulk of the disease present, ranging from less than 10% in the N0 neck to over 70% in patients with positive nodes at multiple levels [3]. The addition of either pre- or post-operative radiation therapy further reduces the incidence of failure in the neck by at least 50% for all N stages [4,5]. However, whilst the RND provided a reliable method of treating patients with head and neck cancer, it became increasingly apparent that it carried substantial morbidity and complications. Nahum described a syndrome of decreased range of abduction in the shoulder joint and pain following RND which has been termed 'shoulder syndrome' [6]. The aetiology of this syndrome is in part sacrifice of the spinal accessory nerve (SAN) with preservation of that structure during neck dissection ameliorating the syndrome [7]. The morbidity of the classical RND thus gave impetus to the development of modified procedures that attempted to reduce the adverse effects of the classical operation and yet preserve its effectiveness in oncological terms.

The realisation that it was possible to perform a complete en-block lymphadenectomy with preservation of structures such as the SAN led to the development of less morbid procedures, the original concept of which is credited to Bocca [8]. The ability to harvest neck nodes in an operation that limits morbidity has led to a more proactive approach to cervical disease with many clinicians advocating elective neck dissections in those patients whose primary site characteristics would suggest a high rate of occult metastasis. Although this has been demonstrated to be of survival benefit, even in sites such as the tongue with rates of occult cervical disease approaching 50% in some series the majority of patients undergoing an elective, staging neck dissection will ultimately demonstrated to be disease-free [9,10]. As such it is imperative that complications of such surgery and their attendant morbidity are minimised.

Morbidity is best minimised by a meticulous approach which begins in the out-patient setting. This discussion will therefore present an evidence-based approach to the care of patients undergoing neck dissection beginning with pre-operative evaluation and peri-operative care prior to consideration of surgical technique itself. Although the ultimate complication of neck dissection is residual or recurrent tumour a logical approach such as this should permit maximum eradication of disease and improve survival whilst minimising hospital stay, complications and even mortality.

## General considerations

Co-morbidities such as cardiac, respiratory and hepatic disease are common place in patients undergoing neck dissections in either an elective or therapeutic sense. Additional immunosuppression caused by conditions such as diabetes or relative malnutrition should be optimised since they predispose to complications including as wound infection.

## Pre-operative evaluation

Reduced nutritional intake may be a direct result of symptoms from the index tumour such as dysphasia or odynophagia and in itself is an independent contributory factor to poor gastrointestinal function. In appropriate cases enhancement of nutritional status with either a percutaneous or radiologically-inserted gastrostomy should be considered since this will not only help to reverse any malnutrition pre-operatively but will also ensure prompt enteral feeding in the post-operative period. Patients undergoing isolated neck dissections rarely require transfusion, but if such surgical approaches are combined with ablation of the index tumour and in particular microvascular free tissue transfer allogenic blood is often indicated with it attendant problems including communicable disease transmission. In prognostic terms the immunosuppressant effects such blood transfusions have not been shown to have a detrimental impact on survival but autogenous transfusion or peri-operative normovolemic haemodilution remain viable alternatives [11,12].

The issue of how long it takes to benefit from a tobacco cessation is not well established but even avoidance for 12-24 hours may be advantageous to the cardiovascular system in terms of carbon monoxide and nicotine elimination. Only rarely do smoking withdrawal symptoms such as agitation and disorientation occur in such circumstances. Cessation of several days greatly improves ciliary flow but post-operative respiratory morbidity is only reduced if tobacco is avoided for a minimum of two weeks. Likewise excessive alcohol use amongst head and neck cancer patients is common with both minor and major post-operative withdrawal symptoms increasing morbidity. Delirium tremens confers appreciable mortality and its prevention should therefore be an achievable goal.

## Anaesthesia

The importance of communication between the anaesthetic and surgical teams cannot be over emphasised. There are theoretical advantages to using low-molecular weight as opposed to standard low dose heparin in the prophylaxis of deep vein thrombosis and pulmonary emboli since it lessens bleeding complications, has a prolonged duration of action and is less likely to induce thrombocytopaenia [13]. Physical methods of prophylaxis including external pneumatic compression and gradient elastic stockings should be universal. Intra-operative hypovolemia is a potential cause of organ dysfunction, increased post-operative morbidity and death. Whilst over-administration of crystalloids can lead to pulmonary oedema, goal-directed volume expansion during the intra-operative period using oesophageal Doppler has been shown to improve not only stroke volume, cardiac output and post-operative recovery but also reduces the time until patients are declared medically fit for discharge [14,15]. The use of LidCo for fluid optimisation in the intensive care setting is well-established. Intra-operative LidCo rapid monitoring, particularly when combined with pre-operative cardiopulmonary exercise testing, may confer similar advantages [16,17].

## Post-operative care

Peri-operative cardiac complications can often be minimised by proactive management, e.g. the use of intra-operative lignocaine on the carotid sinus to counter potential arrhythmias caused by digital manipulation. Early hypertension may follow RND possibly as a result of either carotid sinus denervation or a Cushing's reflex associated with intra-cranial hypertension [17]. If such hypertension is encountered its incidence is increased if the contra-lateral neck is subsequently dissected. Prolongation of the Q-T interval has been reported following right-sided neck dissection and under such some circumstances special attention should be given to potassium levels [18]. Aggressive respiratory support is mandatory in encouraging patients to clear secretions and is especially important in the tracheotomised individual in whom pain may further limit pulmonary function. Early mobilisation further decreases morbidity. If shoulder function is impaired proactive treatment with physiotherapy should be instituted [19].

## Surgical technique

### Asepsis

Although preparation of a surgical site prevents wound contamination by removing transient pathological bacteria and decreasing resident flora counts, good surgical technique with minimal tissue damage still has a role to play. Removal of large amounts of beard hair may increase the rate of infection unless carried out immediately prior to surgery. If concurrent en-bloc resection of a primary oral or oro-pharyngeal lesion is to be carried out the resultant through-and-through defect increases the rate of wound infection in the neck although topical treatments with either antiseptics or antibiotics have been demonstrated to be beneficial [20]. Reducing the length of peri-operative treatment with intra-venous antibiotics limits the development of drug-resistant bacterial infection, e.g. *Methicillin*-resistant *Staphalococcus Aureus *[21].

### Incisions

A variety of approaches exist for the approach to neck dissections that usually simply rely on surgeon preference, e.g. wine glass incision, apron access. The vascular supply of the cervical skin is derived from the external carotid artery superiorly and the subclavian artery inferiorly. Trifurcations or incisions parallel to the carotid artery should be avoided particularly in salvage cases after radiotherapy. In the latter instances some surgeons prefer the security of a McFee incision which avoids some of the potential problems of three-point access.

### Flap elevation and closure

Flaps should be elevated in the sub-platysmal plane in order to maximise their blood supply unless local disease dictates otherwise. Tissue should be incised in stages particularly if bilateral flaps are employed since this limits blood loss. Skin flaps should not be allowed to dry out and if necessary before closure 2-3 mm of the edges should be excised. If a tracheostomy is to be performed it should not be incorporated into the main surgical field since not only may sepsis become a major consideration but also the ability to preserve vacuum for drains at closure may be compromised. Careful approximation of tissues should be a balance between preventing dead space formation on the one hand and causing necrosis by over-tightening on the other.

### Progression of neck dissection

The sequence of the neck dissection is dictated by the type of procedure being undertaken, whether such surgery is being performed simultaneously with other ablative procedures, e.g. thyroidectomy or pharyngolaryngectomy, and ultimately by the preference of the surgeon. In selective neck surgery the first step is often to divide the fascia overlying the sternocleidomastoid muscle. A plane can then be developed around both the anterior and posterior borders of the muscle as required. Dissection at the base of the neck around the anterior aspect of the sternocleidomastoid muscle exposes the omohyoid muscle which is an important landmark. Most surgeons will dissect the upper deep cervical chain from inferior to superior, exposing the internal jugular vein (IJV), carotid system and vagus nerve in so doing. In the upper neck the digastric muscle should be exposed in its entirety and forms another useful landmark. Dissection along the anterior belly brings the surgeon into the midline and directs the dissection to the inferior border of the mandible whereas the hypoglossal nerve and its associated troublesome venous plexus can be identified above the tendon of the muscle. The posterior belly of digastric delineates a safe area lateral to the upper end of the internal jugular vein. If the posterior triangle needs to be addressed it is often best to identify the trapezius muscle in the first instance and then incise parallel to its fibres onto the pre-vertebral fascia. It is important to identify this fascia since it not only protects the underlying neural structures but also provides a plane along which dissection is readily facilitated.

### Air embolus

This is a rare event which can occur following injury to the IJV. Large emboli can produce sudden falls in end-tidal carbon dioxide and arterial blood pressure. A pre-cordial Doppler probe may detect the characteristic murmur of venous embolus. Local pressure should be applied and the anaesthetist informed so the patient can be placed in the Trendelenburg position and rotated to the left. In severe cases attempts can be made to pass a catheter and aspirate air from the right side of the heart. Hyperbaric oxygen therapy, were available, is the ultimate and effective treatment.

### Pneumothorax

This may occur when working low in the neck particularly if the lung apex is high as may occur in over inflation secondary to inadvertent one-lung intubation. Any tears in the pleura should be closed and their integrity tested by hyperinflating the lung, placing the patient in the Trendelenburg position and irrigating the area with clear fluid to observe bubbles. On table imaging may be necessary to determine the need for open chest drainage.

### Chyle leak

The thoracic duct arises from the cisternal chyli at the level of the second lumbar vertebra and rises into the neck between the aorta and the azygos vein. In the thorax it crosses to the left and after passing behind the aortic arch and left subclavian artery it lies on the anterior scalene muscles and phrenic nerve. The duct terminates most commonly in the left IJV although less commonly it may enter the left subclavian, left external jugular, left brachiocepalic (innominate) vein or right IJV. Up to 50% of patients exhibit more than one termination of the duct. The right lymphatic duct drain terminates at the junction of the right subclavian vein and IJV. The key to treatment of a chyle fistula is prevention which demands knowledge of the relevant anatomy. Whereas intra-operative identification can be aided by placing the patient in the Trendelenburg position or adopting a forced Valsalva manoeuvre, post-operative leaks are usually identified when feeding is commenced. Multiple approaches to the treatment of an established leak have emerged including nutritional, surgical and pharmalogical therapy. Although there are strong feelings amongst clinicians about the use of bowel rest, parental nutrition or low fat enteral formulae for the treatment of established chyle leaks, definitive evidence supporting one therapy over another does not exist [22].

### Neural structures

Reparative processes of transected cervical nerves may lead to neuromas in the early or late post-operative period. A number of techniques have been advocated to eliminate such neuroma formation including ligation of the cut edge, cautery, alcohol injection and burying the cut end of nerves into muscle. None of these techniques have any proven value in preventing neuroma.

In limiting neural injury during neck dissection the surgeon arguably focuses most attention on the SAN which can be safely preserved without jeopardising the integrity of tumour exenteration as long as it not grossly involved by disease [23]. Care should be taken when elevating the skin flaps in the posterior triangle as occasionally the SAN lies surprisingly superficially. Consistent and rapid identification of the SAN in the upper neck during dissection that can be facilitated by a number of methods, many of which rely on the identification of Erb's points [24]. The attachment of the sternocleidomastoid muscle to the mastoid process is usually tendonous laterally, the inferior extent of this portion of the muscle often corresponding to the emergent point of the SAN from the medial muscle. A small vessel invariably lies immediately over the SAN in a relationship analogous to the artery that it often encountered whilst exposing the main trunk of the facial nerve. Whilst some authors have suggested cable grafting the SAN if formal resection is undertaken, intra-operative electroneurography and histochemical staining have demonstrated that maintenance of the C2, C3 and C4 branches to the trapezius muscle preserves useful function and can permit en-bloc resection of the proximal part of the SAN with the specimen if oncologically indicated [25]. If significantly reduced shoulder pain and disability is apparent in the early post-operative phase then progressive resistance exercise training should be instituted in an attempt to minimise its impact and improve quality of life [26].

Whereas the ansa cervicalis is frequently sacrificed the vagus, lingual, hypoglossal and marginal mandibular branch of the facial nerves should be identified and preserved. Identification of the latter should be attempted early on in the neck dissection following flap elevation. If the nerve cannot be identified by visual inspection through the layers of the deep cervical fascia its integrity should be preserved by dividing the facial vessels approximately 1 cm below the lower border of the mandible and then retracting the divided ends upwards, so lifting the marginal mandibular branch of the nerve away from the surgical field. The hypoglossal nerve can be identified crossing the external carotid artery and then emerging from underneath the posterior belly of digastric on the hyoglossus muscle - it is at risk of inadvertent damage at both sites. Transient neuropraxia to the phrenic nerve is often manifested sub-clinically in the post-operative period with changes on plain radiography but if a severe pulmonary problem exists, especially with concurrent pectoris major flat harvest, respiration may be compromised. Bilateral phrenic nerve palsies may necessitate periods of prolonged mechanical ventilation. Although the carotid plexus of the sympathetic trunk is at risk of incurring injury the presence of Bernard-Horner's syndrome in the post-operative period is less than 1% [27]. Paralysis of the cranial nerves occurs only rarely (less than 2% of neck dissections) and as such they are difficult to predict but are not associated with non-neurological complications [27].

The brachial plexus lies between scalenus anterior and medius muscles as it crosses the posterior triangle. It is not usually encountered other than in the extended radical neck dissection but knowledge of its location is important in preventing further readily avoidable complications. Intentional transection of the vagus nerve can result in intra-operative cardiac problems of which the anaesthetist needs to be forewarned.

Where possible integrity of the cranial nerves should be maintained unless this compromises tumour resection. Although it is well established that resection of the facial nerve in parotid malignancy provides similar survival benefit to preservation and post-operative radiotherapy, no similar data is available for other cranial nerves with relevance to neck dissection. Nerve resection therefore remains an individual decision and may well be influenced by other matters, e.g. the relatively low threshold for sacrificing a hypoglossal nerve if during the same operation a formal ipsilateral hemi-glossectomy is to be performed.

### Drains

Drainage is used following neck dissection to prevent the collection of fluid and to aid healing. The placing of drainage should be carried out separately from the incision to reduce the risk of infection. Although there is some controversy whether active or passive drains should be used when neck dissections are carried in conjunction with free tissue transfer, the evidence suggests that active drainage should be employed in both free flap and non-free flap cases [28].

### Extended neck dissections

In addition to neural structures, vascular elements may also have to be sacrificed in certain clinically-determined circumstances. Major vessel involvement should be assessed pre-operatively with appropriate imagining, be it contrast-enhanced computed tomography, magnetic resonance imagining, magnetic resonance angiography, ultrasound, Doppler or conventional angiography. Selective sacrifice of the common or internal carotid arteries during extensive cervical operative procedures or their compulsory ligation after exposure for haemorrhage post-operatively can produce some of the most serious complications in head and neck surgery. The highest morbidity occurs in those patients in whom ligation has been performed during a hypotensive episode such as one due to proceeding haemorrhage rather than those in whom selective ligation or excision is undertaken [29]. Balloon-test occlusion with hypotensive challenge offers a simple and reliable method of pre-operative risk assessment when internal carotid artery resection is planned for regional control of disease in advanced head and neck cancer [30]. However, this management option is still associated with a potential for neurological complication that must be weighed against the natural history of the disease and the risk and benefits of other treatment modalities.

## Special considerations

### Bilateral neck dissection

Increased morbidity and mortality has been demonstrated in patients undergoing simultaneous bilateral neck dissections [31]. Higher rates of infections and fistulae occur and complications such as facial oedema and swelling are commonplace, particularly if both IJVs are simultaneous transacted. Raised intra-cranial pressure (ICP) occurs following bilateral IJV ligation with secondary systemic hypertension (Cushing's reflex). This rise in ICP commonly requires aggressive treatment with hyperventilation, fluid restriction, steroids and mannotol. The ICP frequently returns to normal within 24 hours. There can be a significant rise in ICP in a staged second neck dissections even if the subsequent operation is undertaken many years after the initial surgery. Even in cases of bilateral neck dissections where one IJV is preserved post-operative imagining demonstrates thrombosis in up to 30% of cases [32,33] Meticulous technique with prevention of drying of the preserved IJV and minimisation of excess trauma probably reduces the incidence of this occurrence. If both IJVs are to be transected then preservation of conduits in the external venous system should be attempted wherever possible, eg external jugular veins. As in all cases of neck dissection where significant swelling may compromise the airway in the post-operative period the possibility of a prophylactic tracheostomy should be entertained.

### The previously treated neck

Previous treatment of the neck, be it radiation, chemo-radiation or surgery can have a significant impact both in terms of practicalities and post-operative complications. Previous radiation encourages fibrosis between tissue planes such that subsequent dissection can be a laborious process. There is no substitute to a painstaking approach and proactive haemostasis in such circumstances. These problems are often compounded when previous neck surgery has taken place and is of particular issue if vascular access needs to be preserved to form the basis of recipient vessels for free tissue transfer. In such circumstances some of the more commonly used vessels, e.g. facial or superior thyroid arteries, may well have been sacrificed. There is rarely a role for pre-operative arterial imaging but instead the surgeon should be wary of preserving vessels in and around the surgical field. The transverse cervical artery and vein are useful recipient vessels since they are rarely irradiated, are of near constant calibre throughout their course and are not commonly affected by atheroclerosis. In selected circumstances veins grafts may be advisable as an alternative to exposing the contra-lateral neck. In extreme circumstances one may resort to using the cephalic vein turned over the clavicle or the internal mammary system.

### Carotid blow-out

This is associated with over 60% morbidity and 50% mortality. Neurological sequaelae of emergency ligation include hemiplegia, hemi-anaesthesia, aphasia and dysarthria. The incidence is increased following radiation and salary fistulae. Damage to the adventitial layer during surgery may be another contributory factor. If risk of exposure is anticipated vessels should be covered, e.g. dermal graft, fascia lata or levator scapulae muscle flap. This is particularly important in the post-irradiation subject. If impending blow out is suspected (sentinel bleed) endovascular techniques with stent-grafts may be indicated rather than open ligation although short-term complications still occur [34].

## Conclusion

Over the past century the neck dissection has become the accepted face of head and neck oncology, be it performed in isolation or as integral element of a more major resection and reconstruction. Since it is carried out with such regularity in many units it has the potential to be considered as routine. However in common with many procedures the appreciable potential morbidity and indeed morbidity should not be underestimated by junior and seniors surgeons alike. Whilst a meticulous, almost protocol-driven approach should be employed throughout, any one patient's needs can only be truly addressed by an individual approach. Despite the best planning complications can still occur but their impact can be minimised by a vigilant and proactive emphasis in the entire peri-operative period.

## Competing interests

The authors declare that they have no competing interests.

## Authors' contributions

CK Surgical technique

Special considerations

Conclusions

MH Introduction

General considerations and peri-operative evaluation

Both authors read and approved the final manuscript
